# Pattern of novel psychoactive substance use among patients presented to the poison control centre of Ain Shams University Hospitals, Egypt: A cross-sectional study

**DOI:** 10.1016/j.heliyon.2022.e10084

**Published:** 2022-08-12

**Authors:** Ahmed Hashim, Nouran A. Mohammed, AlFadl Othman, Mohab A.K. Gab-Allah, Ahmed H.M. Al-Kahodary, Eslam R. Gaber, Ahmed M. Hassan, Mahmoud Aranda, Rania Hussien, Amany Mokhtar, Md. Saiful Islam, Ka Yiu Lee, Muhammad Sohaib Asghar, Muhammad Junaid Tahir, Zohaib Yousaf

**Affiliations:** a Department of Community, Environmental and Occupational Medicine, Faculty of Medicine, Ain Shams University, Cairo, Egypt; b Department of Forensic Medicine and Clinical Toxicology, Faculty of Medicine, Ain Shams University, Cairo, Egypt; c Department of Public Health and Informatics, Jahangirnagar University, Savar, Dhaka, 1342, Bangladesh; d Swedish Winter Sports Research Centre, Department of Health Sciences, Mid Sweden University, Östersund, Sweden; e Dow University of Health Sciences–Ojha Campus, Karachi, Pakistan; f Lahore General Hospital, Lahore, 54000, Pakistan; g Hamad Medical Corporation, Doha, Qatar; h Centre for Advanced Research, Excellence in Public Health, Savar, Dhaka-1342, Bangladesh

**Keywords:** Novel psychoactive substance (NPS), Intoxication, Strox, Voodoo, Drugs, Addiction, Awareness

## Abstract

**Background:**

Novel psychoactive substances (NPSs) are relatively new substances in the illicit drug market, not previously listed in the United Nations Office on Drugs and Crime (UNDOC). Strox and Voodoo are considered some of the most popular blends of NPS in the Egyptian drug market.

**Objectives:**

The current study was conducted to assess NPS's use pattern: Voodoo and Strox among acutely intoxicated patients presented to the poison control center of Ain Shams University Hospitals (PCC- ASUH).

**Methods:**

A single center based cross-sectional study was carried out in the PCC-ASUH among acutely intoxicated patients presenting to the emergency department (ED) over four months (from January–April 2019. using a previously adopted and validated Fahmy and El-Sherbini socioeconomic scale (SES). Data were presented as mean, median and range as appropriate. Both smoking and crowding indexes were calculated and presented as previously reported.

**Results:**

Fifty-one patients were presented to the ED of PCC-ASUH during the study period. A total of 96.1% (n = 49) were males. The mean age was 25 ± 7.5 years. The most common NPS used was Strox: 54.9% (n = 28), followed by Voodoo: 27.4% (n = 14). Neurological and gastrointestinal (GI) symptoms were the most frequent presentations. The most common motive behind NPS use was the desire to give a trial of new psychoactive substances. The mean SES score was 35.1 ± 13.17. Most patients have the preparatory as the highest education 36.0% (n = 18).

**Conclusions:**

NPS use is common among young males in preparatory education from different social classes, starting it most commonly as a means to experiencing a new high. Neurological and GI manifestations are the most common presenting symptoms of NPS intoxication.

## 1 Introduction

The European monitoring center for drugs and drug addiction (EMCDDA) defines "novel psychoactive substance" (NPS) as any new narcotic or psychotropic drug either in pure form or as preparation not under the control of the United Nations Office on Drugs and Crime (UNODC) that threaten public health compared to a substance listed in conventions (EMCDDA, 2019). United Nations (UN) classifies NPS according to similarities in the chemical structure into seven groups. These groups are synthetic cannabinoids, synthetic cathinones, ketamine, phenethylamines, piperazines, plant-based substances and miscellaneous substances (UNODC, 2013) Undetermined mixtures with similar names can differ in constitution from one country to another and even between two packages. This can lead to inaccurate dose calculation and hazardous adulterants (Bogusz, 2017; Zamengo et al., 2014). This, in turn, can result in a wide range of physical symptoms which can be life-threatening (Tracy et al., 2017).

There is a notable difference in the chemical structures between NPS and known illicit substances. Those differences in structure may lead to difficult detection by toxicological screening methods. This is why these substances are referred to as "legal high" (Cohen and Weinstein, 2018; ElSohly et al., 2014; Tang et al., 2019; Zamengo et al., 2014). It is a growing problem despite the difficulty of determining the accurate prevalence of NPS use (Carter et al., 2009; Madras, 2017). United States (US) has reported increasing synthetic cannabinoids (SC) use-related intoxication and death. 1.3% of secondary school students in the US use NPS (Trecki et al., 2015; UNODC, 2017). Despite the measures taken by UNDOC, NPS abuse continues to increase (Dargan and Wood, 2012). NPS market has expanded globally in recent years. In 2009, about 130 substances were available, increasing to 479 NPS by 2016 (UNODC, 2018). Many factors affect the dramatic increase of NPS abuse, such as its ease of availability at street dealers and the internet, especially social networks (Miliano et al., 2018).

Strox and Voodoo are considered two of the most popular blends of NPS in the Egyptian drug market. These NPSs are usually made in clandestine laboratories with substantial variation in the types and concentrations of chemical constituents and adulterants, further contributing to the variation in clinical presentation (Hussien et al., 2021). They have also demonstrated that sample Voodoo NPS has a broad spectrum of various previously identified bioactive compounds, including quinazolines, morphinan alkaloid, cannabinoids, penitrem A and the well-known synthetic cannabinoid FUB-AMBcn (Hussien et al., 2020). Their chemical analysis has also revealed the presence of common psychoactive compounds such as tetrahydrocannabinol (THC), amphetamine, 3,4- methylenedioxyamphetamine, tramadol and oxazepam. Notably, there is an increase in the rate of Strox use up to 20.7% in 2018 compared to 2% in 2017 (FDCTA, 2018). Additionally, a recent national health survey conducted among secondary school students has revealed that 14.9% and 12.4% of students use Strox and Voodoo, respectively (FDCTA, 2017). The Poison Control Center of Ain Shams University Hospitals (PCC-ASUH) received 361 Strox and 125 Voodoo intoxicated patients in 2018 (PCC-ASUH, 2018). The point prevalence of NPS abuse among 5 non-medical colleges in Egypt was previously estimated to be 6.8% (Hashim et al., 2020). This study aimed to assess the pattern of NPS (Voodoo and Strox) use among patients presented to the PCC- ASUH. Other secondary outcomes were directed to the socio-demographic determinant of the detected pattern of abuse.

## 2 Methodology

### 2.1 Study design and patient population

This single-center based cross-sectional study was conducted at the emergency department (ED) of the PCC-ASUH. PCC-ASUH specializes in dealing with acute and chronic intoxicated patients with different modes of exposure, either accidental or suicidal, in addition to patients with drug overdoses. It is one of the six toxicology centers all over Egypt and one of 2 centers in Cairo, according to the Ministry of Health and Population (MOHP). The center receives an average of 20,000 intoxicated patients every year (MOHP, 2019). Criteria for inclusion were: 1) Patients with acute NPS intoxication (Voodoo, Strox). 2) Patients with acute intoxication with any other substances of abuse together with a history of current NPS abuse. Criteria for exclusion were: 1) Patients with altered mental state who couldn’t be interviewed. 2) Participants who didn’t provide consent or withdrew the consent. 3) Patient with no history of NPS use.

Intoxication was defined as a state of altered consciousness, hemodynamic abnormalities or abnormal behavior following psychoactive substances consumption (Orsini et al., 2017). The current abuser was defined as any patient who abused NPS in the last 30 days before presentation (Hohmann et al., 2014; Wonguppa and Kanato, 2018). Delay time was defined as the time between drug administration and ED presentation (Parasuraman, 2011). The endpoint of the study was either discharge or death of the patient.

### 2.2 Variables and data collection

Data were collected by eight interviewers using the convenience sampling method. All the interviewers were dedicated to covering the ED for 24 h over seven days from January 2019 to April 2019. Data were collected using a semi-structured interview questionnaire previously developed by Fahmy and El Sherbini (1983) in Arabic to assess family socio-economic status (EL-Sayed Hossein and Mostafa Almarhomy, 2017; Fahmy S, 1983). The scale was further extended and updated by El-Gilany and El-Wehady translated into Arabic (Cronbach’s alpha = 0.66) (El-Gilany et al., 2012). All the interviewers were trained on the translated version to avoid interviewer bias. After the translation, the Arabic version was distributed to public health and toxicology experts to ensure that the questionnaire language and content was simple and comprehensive. Additionally, a pilot study was conducted on six patients to test and establish the validity of the questionnaire which has yielded some modifications resulting in the final version of the questionnaire. The questionnaire comprised three sections; the first section, the ED clinical data on presentation, abused substances, delay time, symptoms, combined substances, previous presentation to the ED and patient's outcome. The second section, NPS abuse history, history of other abused substances, reason for NPS abuse and any effort to quit. The third section, socio-demographic and socioeconomic data. Socioeconomic data included "updated Fahmy and El-Sherbini socioeconomic scale (SES)” previously used in health research in Egypt (Abbass et al., 2019; Abbass et al., 2019; El-Gilany et al., 2012). SES comprised the educational, occupational, family, family procession, home sanitation, economic and health care domains. The Cronbach’s alpha of updated Fahmy and El-Sherbini socioeconomic scale (SES) applied in the studied population was 0.543.

### 2.3 Ethical considerations

Data was collected after obtaining verbal informed consent just before discharge when the patient was in a complete optimal mental and physical condition that permitted him/her to be discharged. For patients with an age less than 18 years old, the consent was taken from the attending caregiver. The ethical approval was granted by the Faculty of Medicine, Ain-Shams University Research Ethics Committee and administrative approval was obtained by the PCC-ASUH.

### 2.4 Statistical analysis

Statistical analysis was carried out using a statistical package for the social sciences (SPSS) version 24.0. Determination of the social class was carried out by interquartile calculation range as previously postulated by the original SES (El-Gilany et al., 2012). The crowding index was defined as the total number of co-residents per household, excluding the newborn infant, divided by the total number of rooms, excluding the kitchen and bathrooms (Melki et al., 2004). The smoking index was defined as a unit for measuring cigarettes consumption over a long period. It was previously calculated using the following formula: smoking index = CPD × years of tobacco use (Nagata et al., 2013; Nance et al., 2017).

## 3 Results

**Figure 1:**
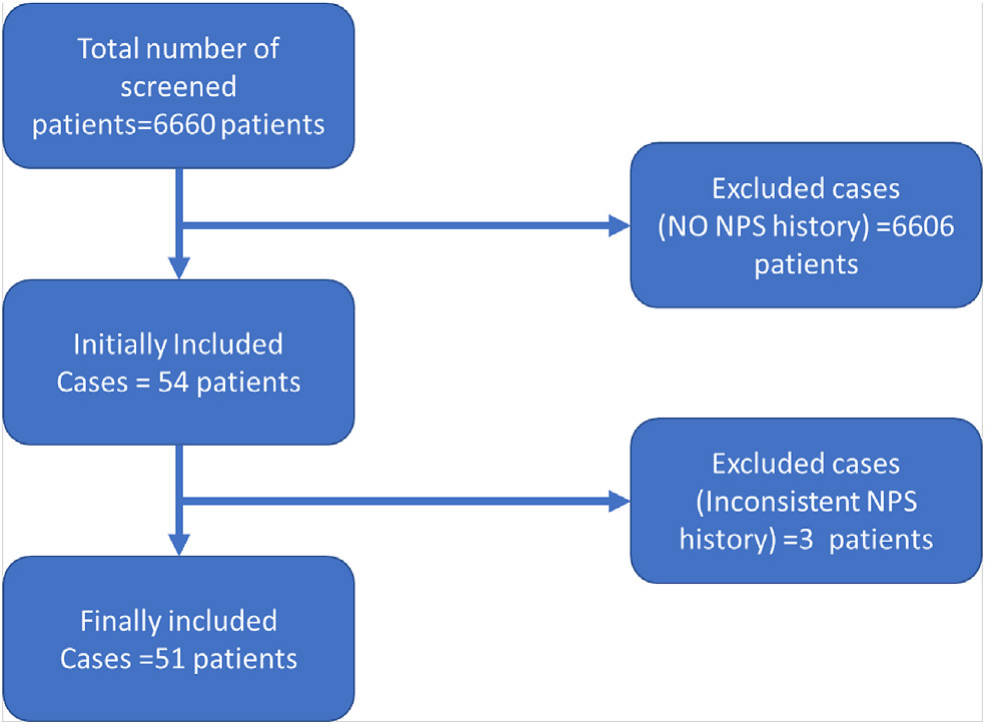
Flow chart of the patient inclusion and exclusion.

Fifty-one patients, who met the inclusion criteria, were enrolled in the current study. Three patients were excluded because of their inconsistent history of NPS abuse (Figure 1). Most of the studied patients were males (n = 49, 96.1%) with a mean age of 25 ± 7.5 years. Thirty-six patients were single (72.0%) (Table 1).

**Table 1:** Socio-demographic data of the studied patients.

Demographic data	
**Gender (n = 51)**	**n (%)**
*Male*	49 (96.1)
*Female*	2 (3.9)
**Age (n = 49)**	***n (%)***
***<****18 years*	5 (10.2)
*18-28 years*	34 (69.4)
***>****28 years*	10 (20.4)
Mean ± SD	25 ± 7.5
**Marital Status (n = 50)**	**n (%)**
*Single*	36 (72.0)
*Married*	12 (24.0)
*Divorced*	2 (4.0)
**Level of education (n = 50)**	**n (%)**
*Illiterate*	8 (16.0)
*Read and write*	2 (4.0)
*Primary education*	5 (10.0)
*Preparatory education*	18 (36.0)
*Secondary education*	9 (18.0)
*Intermediate education*	5 (10.0)
*University graduate*	3 (6.0)
**Domains of SES**	
*Educational Domain*	
Mean ± SD	10.8 ± 8.6
Median (Range)	10 (0–28)
*Occupational domain*	
Mean ± SD	2.7 ± 2.3
Median (Range)	2 (1–9)
*Family domain*	
Mean ± SD	5 ± 1.6
Median (Range)	5 (2–10)
*Family-procession domain*	
Mean ± SD	4.9 ± 1.9
Median (Range)	5 (0–9)
*Health sanitation domain*	
Mean ± SD	7.67 ± 2.1
Median (Range)	7 (3–11)
*Economic domain*	
Mean ± SD	1.14 ± 1.06
Median (Range)	1 (0–4)
*Health care domain*	
Mean ± SD	3 ± 1.05
Median (Range)	3 (1–5)
*Total SES*	
Mean ± SD	35.1 ± 13.17
Median (Range)	32 (12–63)
Social class (n = 49)	n (%)
Very low	15 (30.6)
Low	10 (20.4)
Middle	12 (24.5)
High	12 (24.5)
Patient occupation (n = 50)	n (%)
Not working	11 (22)
Unskilled manual worker	26 (52)
Skilled manual workers	10 (20)
Trades	3 (6)
**Crowding index (n** = **49)**	**n (%)**
>1 person room	28 (57.1)
= 1 person per room	21 (42.9)
**Access to health services (n** = **48)**	**n (%)**
*Traditional or self-health care Free*	3 (6.3)
*Governmental health services*	26 (54.2)
*Health insurance*	2 (4.2)
*Private health facility More than*	7 (14.6)
*one of the above accesses*	10 (20.8)

SD: Standard deviation SES: updated Fahmy and El-Sherbini socioeconomic scale.

The educational status was ranging from illiterate in 8 patients (16%), read and write in 2 (4.0%), primary education in 5 (10.0%), preparatory education in 18 (36.0%), secondary education in 9 (18.0%), intermediate education in 5 (10.0%) and university graduate in 3 (6.0%). Their occupational status was: eleven patients (22.0%) were not working, 26 (52.0%) were unskilled manual workers, 10 (20.0%) were skilled manual workers while 3 (6.0%) were petty traders (Table 1).

"Updated Fahmy and El-Sherbini SES" was used to speculate the socioeconomic level of the admitted patients. The mean score of SES was 35.1 ± 13.17. SES revealed the following results: the mean of the educational domain was 10.8 ± 8.6, occupational: 2.7 ± 2.3, family: 5 ± 1.6, family procession: 4.9 ± 1.9, health sanitation: 7.67 ± 2.1, economic: 1.14 ± 1.06 and health care: 3 ± 1.05 (Table 1).

The social class of studied patients ranged from very low (15, 30.6%), low (10, 20.4%), middle (12, 24.5%), and high class (12, 24.5%) (Table 1).

The most frequently encountered clinical manifestations were neurological and gastrointestinal (GI) manifestations with considerable overlapping of symptoms in some patients. Out of 45 patients, 18 (35.29%) presented mainly gastrointestinal manifestation. Neurological manifestations accounted for 35.29% of the presenting symptoms in the studied population. The neurological presentations included agitation, hallucination, generalized numbness, coma, convulsions and slurred speech. GI manifestations included diarrhea, vomiting and abdominal pain. Cardiopulmonary manifestations were the least encountered type of presentation, with 9 patients (17.64%) presented with dyspnea, palpitation and chest pain (Table 2). Patients who met the inclusion criteria were categorized into two groups: 37 (72.6%) were regular NPS abusers and 14 (27.4%) were first trial NPS abusers (Table 2).

**Table 2:** Clinical manifestations, Patient category, and outcome of studied patients.

**Clinical manifestations (n = 45)**	**n (%)**
GIT manifestation (e.g., vomiting, abdominal pain …)	18 (35.29)
Neurological manifestation (e.g., agitation. hallucination, etc.)	18 (35.29)
Cardiac manifestations (e.g. chest pain, palpitation ….)	9 (17.64)
**Patient Category (n = 51)**	**n (%)**
Current NPS abusers and presented with NPS intoxication	28 (54.9)
First trial NPS abusers and presented with NPS intoxication	14 (27.4)
Current NPS abuser and presented with other illicit substances intoxication	9 (17.7)
**Patient outcome (n = 47)**	**n (%)**
Observation and discharge	30 (63.8)
Admission to the in-patient unit	5 (10.7)
Admission to ICU	8 (17.0)
Referral to another hospital	4 (8.5)

The most commonly intoxicated NPS was Strox; 28 (54.9%) followed by Voodoo; 14 (27.4%). Illicit substances other than Voodoo or Strox (e.g., tramadol, cannabis, benzodiazepine, opiate, alcohol) were the causes of the presentation in 9 current NPS users (17.7%). The clinical outcome showed that out of the 47 patients seen, 30 (63.8%) observed then discharged after a 6-hours surveillance system (CHEMM, 2021), 8 (17%) were admitted to the ICU, 5 (10.7%) were admitted to the in-patient unit and 4 (8.5%) were referred to another hospital (Table 2). The pattern of abuse varied amongst the patients. Out of 48 patients seen, 22 (45.8%) had history of daily abuse while 14 (29.2 %) were first time trials (Table 2). The reported triggers to start NPS abuse (Strox or Voodoo) varied. Twenty-five patients (55.6%) reported that the trial of a new high was the trigger to start using NPS (Table 3).

**Table 3:** Pattern of NPS (Strox and Voodoo) use.

**The pattern of NPS abuse (n = 48)**	**n (%)**
Trial	14 (29.2)
Yes, but not regular.	8 (16.7)
Current (1–2) days/week	3 (6.3)
Current (5–6) days/week	1 (2.1)
Daily	22 (45.8)
**Number of cigarettes smoked each day (n = 30)**	
Mean (SD)	7.28 (11.14)
Median (Range)	4 (0.5–60)
**How long being regularly in years (n = 28)**	
Mean (SD)	2.46 (2.62)
Median (Range)	1 (0.16–10)
**Number of Egyptian pounds spent per week (n = 24)**	
Mean (SD)	389.58 (405.88)
Median (Range)	235 (35–1500)
**Methods of NPS obtaining**∗	**n (%)**
Local dealer	25 (53.2)
Friends	25 (53.2)
Other sources (e.g., Parties)	3 (6.4)
**Reasons for NPS use**∗	**n (%)**
Try a new high	25 (55.6)
Friend using it	15 (33.3)
Cheaper than cannabis	14 (31.1)
Easy to obtain	12 (26.7)
Depression	8 (17.8)
I could not get cannabis	7 (15.6)
Accidental	6 (13.3)
Similar cannabis high	4 (8.9)

SD: Standard deviation NPS: Novel Psychoactive Substance.

∗ Overlap of Items is possible.

Most of the participants (87.5%) were daily tobacco smokers. Of the 48 patients, two-thirds (66.7%) had a history of cannabis smoking with different smoking patterns: 10 patients were not regular users (20.8%), 9 (18.8%) were regular users 2–3 times/week and 13 (27.1%) were daily users (Table 4). Alcohol was the second most commonly abused substance with 16 patients (33.3%) of the studied patients (48 patients) were reported alcohol users (Table 4).

**Table 4:** Other illicit substances abused with NPS among studied patients.

**Tobacco smoking status**∗	**n (%)**
Cigarette Smoking	42 (87.5)
Shisha Smokers	9 (19.1)
**Cannabis abuse (n = 48)**	**n (%)**
None users	16 (33.3)
Not Regular user	10 (20.8)
Regular 2–3 times/week	9 (18.8)
Daily	13 (27.1)
**Tramadol abuse (n = 48)**	**n (%)**
None users	37 (77)
Not Regular user	5 (10.4)
Regular 2–3 times/week	3 (6.3)
Daily	3 (6.3)
**Alcohol abuse (n = 48)**	**n (%)**
None users	32 (66.7)
Not Regular user	14 (29.1)
Regular 2–3 times/week	1 (2.1)
Daily	1 (2.1)

∗ Overlap of Items is possible.

## 4 Discussion

NPS abuse is a significant global problem. The NPS are characterized by the ambiguity of its chemical constituents resulting into wide range of adverse events. A better understanding of the epidemiological, biological, toxicological and clinical aspects is needed to overcome the growing use of NPS (Madras, 2017). This study aimed to investigate the pattern of NPS use with its socio-demographic and socioeconomic correlates of patients attending PCC-ASUH.

The number of included patients was comparably similar to previous studies investigating clinical parameters of NPS as a cause of ED presentation or ICU admission (Kabata et al., 2015; Orsini et al., 2017). Most of the patients (54.9%) presented with abuse of herbal mixture known as Strox. However, 27.4% of patients abused other herbal mixtures known as Voodoo, with the remaining patients (17.7%) being current NPS abusers and presenting due to other illicit substances of abuse. Several studies have reported using herbal mixtures, which fit the definition of NPS (de Matos et al., 2018; Kabata et al., 2015; Miliano et al., 2018). Occasionally NPS are sprayed on pharmacologically inactive plants, which may be the reason for the term "herbal high" (Hohmann et al., 2014). Voodoo and Strox were the only suspected NPSs recorded during the dedicated study period which creates a sharp contrast with other published scientific data that usually record three or more different NPS-related substances (Bennett et al., 2017). The reasons for the few NPSs observed in this study could be related to restricted access to NPSs information sources (internet and mobile applications) as most respondents admitted to that and showed that they obtained their NPSs either from friends or local dealers (Miliano et al., 2018).

The mortality and discharge data were similar to the previously reported data of acutely intoxicated NPS in Switzerland (Liakoni et al., 2016). Liakoni et al. have reported that most patients (76 %) were discharged home in comparison to 63.8% reported in our study.

A significant heterogeneity has been recorded in the total money spent per week when controlled with the same regular weekly pattern. This significant difference among the same pattern of abuse may reflect a state of massive adulteration (Baumann and Volkow, 2016; Giles Stephenson, 2014; Solimini et al., 2017). The NPS adulteration hypothesis is supported by cholinergic toxidrome and low pseudo choline esterase level after NPS use. Many reasons have been proposed as triggers of NPS abuse; however, "trial of a new high" was the leading cause in agreement with the behavioral philosophy of substance addiction (Grant et al., 2010).

The socioeconomic and socio-demographic factors are generally believed to be detrimental factors in the predisposition of any individual to substance abuse (Diala et al., 2004; Swendsen et al., 2009). The educational status of our patients ranged from illiterate to university graduate. However, most of our patients had the highest educational status of preparatory degree. The educational status has been previously strongly associated with NPS use (Wonguppa and Kanato, 2018). Unemployment is another important detrimental factor causing an increase in NPS use (Wonguppa and Kanato, 2018). However, most of our patients were unskilled manual workers, which may raise the question, "Is it related only to employment or unemployment status?" or the nature of occupation may play a more significant role. Multiple studies have demonstrated the role of specific occupations on the predisposition to substance and alcohol misuse. Three of these occupations are considered unskilled manual works (Coggon et al., 2010; van der Beek, 2014).

Both socioeconomic and socio-demographic factors interact to create the individual's social class. The social class of our patients tilts slightly towards the low social class. More than half of our patients purchased NPS from the same region of residence. The association between the toxic neighborhood and the risk of substance abuse has been previously shown, which may be attributed to the easy accessibility of NPS in these regions, which is considered one of the major factors that create an endemic neighborhood of illicit substance abuse (Mennis et al., 2016; Zhao et al., 2013).

Our study has some inherent limitations, including convenience sampling, small sample size, lack of comparable data on the NPS under study and a lack of a valid addiction questionnaire adapted to the Egyptian society. Another considerable limitation is the barrier related to missing data that may be attributed to the sensitivity of this information and incompleteness of health records. A further prospective study should be warranted to verify these findings.

## 5 Conclusions

NPS use (Voodoo and Strox) is common among young males with preparatory education who are poly-substance abusers and, tobacco smokers from different social classes which normally start as the trial of a new high. Neurological and GI manifestations were the most common presenting symptoms among NPS intoxicated patients. This study highlights the need to raise awareness about the health risks of using these substances, especially amongst pre-university and university students.

## Declarations

### Author contribution statement

Ahmed Hashim: Conceived and designed the experiments; Performed the experiments; Analyzed and interpreted the data; Contributed reagents, materials, analysis tools or data; Wrote the paper.

Nouran A. Mohammed; AlFadl Othman; Mohab A. K. Gab-Allah; Ahmed H. M. Al-Kahodary; Eslam R. Gaber; Ahmed M. Hassan; Mahmoud Aranda; Rania Hussien; Amany Mokhtar; Saiful Islam, MD.; Ka Yiu; Lee, Ph.D.; Muhammad Sohaib Asghar; Muhammad Junaid Tahir & Zohaib Yousaf: Analyzed and interpreted the data; Contributed reagents, materials, analysis tools or data; Wrote the paper.

### Funding statement

This research did not receive any specific grant from funding agencies in the public, commercial, or not-for-profit sectors.

### Data availability statement

Data will be made available on request.

### Declaration of interest’s statement

The authors declare no conflict of interest.

### Additional information

No additional information is available for this paper.
